# Preliminary Data of the Nutritive, Antioxidative, and Functional Properties of Watermelon (*Citrullus lanatus* L.) Flour and Seed Protein Concentrate

**DOI:** 10.3390/molecules30010181

**Published:** 2025-01-05

**Authors:** Agata Hahn, Justyna Liszka, Julia Maksym, Agnieszka Nemś, Joanna Miedzianka

**Affiliations:** 1 Department of Biotechnology and Food Microbiology, Wroclaw University of Environmental and Life Sciences, 37 Chelmonskiego Street, 51-630 Wroclaw, Poland; 121185@student.upwr.edu.pl (A.H.); 121244@student.upwr.edu.pl (J.L.); 121103@student.upwr.edu.pl (J.M.); 2 Department of Food Storage and Technology, Wroclaw University of Environmental and Life Sciences, 37 Chelmonskiego Street, 51-630 Wroclaw, Poland; agnieszka.nems@upwr.edu.pl

**Keywords:** watermelon seed flour, protein concentrate, nutritional properties, antioxidant properties, functional properties

## Abstract

The growing interest in a plant-based diet leads to the search for new sources of protein in the human diet as an alternative to animal proteins. Plant materials that can supplement protein as additives in food products are being studied. Watermelon seeds (*Citrillus lanatus* L.) are rich in proteins and waste from the food industry; however, their extraction is not completely cost-free, and the flour production process may involve additional costs related to their extraction and processing. The studies showed that watermelon seed protein concentrate, obtained using the alkaline extraction method, contained 82.52 g/100 g of protein and 1.51 g/100 g of fat. The polyphenol content in the protein preparation from defatted watermelon seeds was 1.9 mg gallic acid/g, and the antioxidant activity of the concentrate was 29.26 µmol Trolox/g (by the ABTS+). The obtained watermelon seed protein concentrate was characterised by solubility of more than 80% (at pH = 10), water absorption at the level of 2.46 (g water/g) and oil absorption equal to 2.1 (ml oil/g), showed poor foaming properties (1.51%), and was characterised by low emulsification.

## 1. Introduction

Watermelon (*Citrullus lanatus* L.), a member of the Cucurbitaceae family, is one of the plant species commonly used as human food [1]. Watermelon originates from South Africa, and its cultivation has been widespread in regions around the world [2]. According to available data, the annual global production of watermelon is about 100 million tons [3]. The world leaders in watermelon production are China, Iran, Turkey, and Brazil [3]. Watermelon is a horticultural plant grown mainly for its sweet and juicy fruit, which consists of 68% pulp, about 2% seeds, and 30% peel. In terms of chemical composition, watermelon fruit is a source of many nutrients, i.e., proteins, fats, carbohydrates, minerals (potassium, magnesium), and vitamins (mainly vitamin C) [4,5]. However, more than 90% of the total mass of the fruit is water [6].

Watermelon is used in the food industry to produce juices, cocktails and nectars, and its processing generates significant amounts of unused peel and seeds, which are generally treated as waste. The peel is used to produce products, such as pickles and preserves and to extract pectin [7]. Taking into account FAO data from 2022 [3], the estimated waste from watermelons in the form of seeds is from two to five million tons. Watermelon seeds may have potential use in the food industry because when pressing fruit juices, they remain intact after removing the pulp and peel. In recent years, watermelon seeds have become an innovative raw material used in the oil industry [8]. However, they are most often an additive used to feed farm animals due to their rich chemical composition [9]. Watermelon seeds contain an average of 42% fibre, 27% fat, 17% protein, 12% carbohydrates, and 2% ash, and are also a source of minerals, including calcium, phosphorus, potassium, magnesium, sodium, and zinc [10]. In addition, watermelon seeds are a source of polyphenols and, therefore, have antioxidant properties, and their addition to baked goods can increase their health benefits [11] or flour obtained from them can be a substitute for wheat flour in bakery products, including in cookies [12] or bread [13].

The dominant protein in watermelon seeds is globulin, which makes up 50–60% of the total protein content and is soluble in salt solutions. Smaller amounts of albumins, glutelins, and prolamins are also present [14]. The molecular weight of the polypeptides ranges from 35 to 47 kDa, and their isoelectric point falls between pH 4.5 and 5.0 [15]. In addition to traditional extraction methods like alkaline, acidic, and salt extraction [9,16,17,18,19,20,21,22], alternative techniques such as microwave extraction, ultrasound, and enzyme-assisted extraction have also proven effective. These methods typically require an alkaline environment, which can cause protein denaturation, affecting its nutritional value and functional properties [23]. The alkaline extraction method used in this study is efficient, cost-effective, and does not require specialised equipment. It yields a similar amount of protein in less time than other methods. Extraction conditions were optimised using the response surface method of Wani et al. [24], which found the highest yield (80.71 g/100 g) with parameters of 0.12 g/L for the base concentration, a 15 min extraction time, a 70:1 solvent-to-raw material ratio (*v*/*w*), and a temperature of 50 °C. To reduce solvent use, we adjusted these conditions by lowering the solvent-to-raw material ratio to 50:1 (*v*/*w*), decreasing the temperature, and increasing the extraction time to 30 min. A key aim of this study is to preserve the protein’s functional activity and physicochemical properties through a protective extraction medium.

Proteins from seeds are among the top 10 global markets for functional food production, valued for their essential amino acids and health-promoting properties [25]. Watermelon seed proteins, with their rich amino acid profile and nutritional value, are an attractive raw material for food products such as bread, cakes, protein bars, and beverages. As a plant-based protein sourced from waste, it offers an alternative to animal proteins, especially for vegan and vegetarian diets. However, factors like pH, temperature, ionic strength, solvent type, extraction time, and the solid–liquid ratio can complicate industrial-scale extraction [21]. Polyphenols in watermelon seeds may also enhance their antioxidant properties, making them valuable in functional foods [21]. This study aimed to isolate watermelon seed proteins and assess their nutritional, antioxidant, and functional properties.

## 2. Results and Discussion

### 2.1. Chemical Composition

The chemical composition of the analysed watermelon seed flour and protein concentrate are presented in Table 1. The defatting process affected the content of dry matter, total protein, fat and carbohydrates in the flour obtained from watermelon seeds. An increase in the total protein content by 8% and a decrease in the fat content by 88% were noted. The increase in the protein content in the flour resulted from the defatting process, which concentrates the protein by reducing other components, such as fatty acids. The presented data were similar to the results obtained by El-Adawy and Taha [10], analysing defatted watermelon seed kernels. The protein content of defatted watermelon seed flour is lower than in soy and sunflower flours (50–53%) [20]. However, watermelon seeds and the protein preparations obtained from them can be a source of protein in some food recipes. The ash content in both analysed flours was statistically insignificant and ranged from 3.53 to 3.67 g/100 g.

The protein preparation obtained from defatted watermelon seed flour contained over 82 g/100 g of protein in dry matter, 2.50 g/100 g of ash, and 1.51 g/100 g of fat. In the study conducted by Gadalkar and Rathod [19], the protein preparation obtained from defatted watermelon seed flour contained 54.48 g/100 g protein, which is a much lower value compared to that obtained by the authors in the present study but was characterised by a higher content of both fat (4.6 g/100 g) and ash (5.9 g/100 g).

### 2.2. Amino Acid Composition

Based on the amino acid profile of the tested samples (Table 2), it can be stated that the defatting process of watermelon seed flour did not statistically affect the amino acid content. Among the exogenous amino acids, leucine, phenylalanine, and valine were found in the largest amounts (on average 13.92 mg/g, 10.44 mg/g, and 9.12 mg/g, respectively). On the other hand, aspartic acid, glutamic acid, and arginine were found in the largest amounts among all the amino acids analysed (18.86 mg/g, 54.87 mg/g and 25.53 mg/g, respectively). The high content of arginine in watermelon seed flour indicates the therapeutic value of this raw material, and its content is higher than in most other oil seeds, including soybeans and peanuts [20]. The obtained amino acid profiles are consistent with the data presented in the literature. The dominant amino acids in watermelon seeds are mainly arginine, glutamic acid, aspartic acid, and serine [10,21].

More than three times more amino acids, compared to the analysed flours, were found in the obtained protein concentrate (730.69 mg/g). Among the indispensable amino acids, the dominant ones were leucine (50.52 mg/g), phenylalanine (43.01 mg/g), and valine (33.42 mg/g). In turn, almost 50% of all analysed amino acids were aspartic acid (71.71 mg/g), arginine (99.73 mg/g), and glutamic acid (184.83 mg/g). Wani et al. [21] also reported the dominance of glutamic, aspartic acid, and arginine in watermelon protein isolates.

Compared with the FAO-recommended amino acid formula (Table 3), the analysis of the chemical score indices (CS) and essential amino acid index (EAAI) for watermelon seed flour and protein concentrate indicates clear differences in the amino acid profile of the tested samples. The protein concentrate was characterised by higher CS values for most amino acids, including the leucine (103.73%), isoleucine (117.00%), and phenylalanine-tyrosine pair (215.00%), resulting in the highest EAAI (109.46%) compared to non-defatted flour (63.18%) and defatted flour (56.69%). Lysine was the most limiting amino acid (48.44%) in the analysed protein concentrate. Longe et al. [26] also reported lysine and threonine as limiting amino acids in pumpkin (*Telfaria occidentails*) based on amino acid scoring. Low levels of lysine have been reported previously in melon seed proteins [10,21].

### 2.3. Total Phenolic Content and Antioxidant Activity

**Figure 1:**
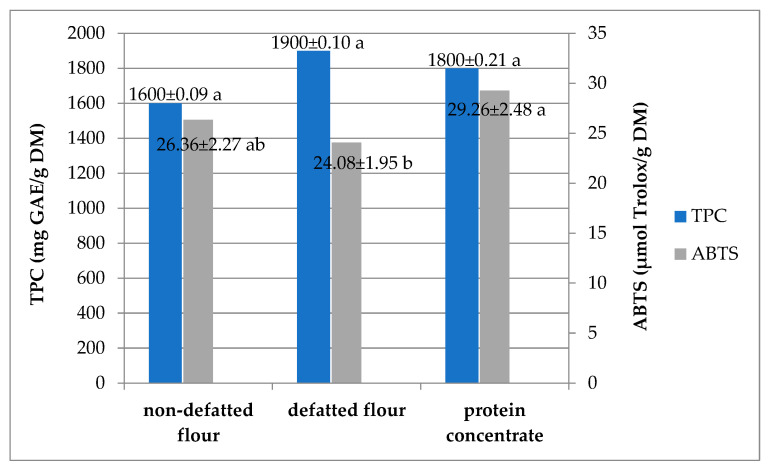
Total polyphenol content (TPC) and antioxidant activity (ABTS+) in the watermelon seed flour and protein concentrate; the same letters within the same analysis indicate values that are not significantly different (Duncan’s test *p* ≤ 0.05 and n = 2).

Phenolic compounds are secondary metabolites naturally present in vegetable material and have been associated with their antioxidant and antimicrobial properties. The total phenolic content (TPC) of two types of watermelon flours and their protein preparation ranged from 1.60 to 1.90 mg gallic acid equivalent (GAE)/g, and this was not statistically significant (Figure 1). The antioxidant activity, determined based on the 2,2′-azino-bis-3-ethylben-zothiazoline-6-sulphonic acid (ABTS˙+), ranged from 24.08 (defatted flour) to 29.26 µmol Trolox equivalent antioxidant capacity (TEAC)/g (protein concentrate). The ABTS˙+ test provides valuable preliminary data on antioxidant capacity, but to gain a more comprehensive understanding, additional tests such as DPPH and FRAP, which assess different antioxidant mechanisms, should be conducted. The TPC data of *Citrullus lanatus* L. seeds are quite common in the literature, but its range is unsurprisingly very wide: 0.04–54 mg GAE/g. This large discrepancy in results found in the literature may be partly due to the variability of the plant material, including the dependence on genetic and cultivation factors, but the different variations in the research methodology used should also be considered. Generally, flour made from so-called high-value seeds (such as cucurbit seeds) has higher levels of phenolic substances (from 1000 mg/kg to as much as 4500 mg/kg) than whole-grain cereal flour [11], including a higher proportion of flavonoids, which have been attributed comparable or even better antioxidant and anti-inflammatory effects than phenolic acids. Therefore, these seeds can be a valuable addition to complement the composition of phenolics in refined flours and should be included in the diet as much (or even more) as whole-grain flour. The watermelon seed protein preparation showed the highest antioxidant activity despite the lack of statistical differences in the total polyphenol content. This indicates that not only the TPC but also the presence of vitamins, flavonoids, and peptides with antioxidant activity can affect the level of antioxidant activity. The content of vitamins C and E can significantly affect the higher values as they also react with the ABTS reagent, giving the same effect as phenolic compounds. The antioxidant activity of the obtained protein preparation makes it not only a good source of protein but also a component with potential health benefits associated with the presence of antioxidants, and these values are comparable to those observed in hazelnut and pumpkin seed flour, which additionally increases its value as a healthy dietary supplement. Nevertheless, the activity of the analysed flour and protein concentrate was relatively low, which was probably due to the low level of total phenolic compounds, which are usually involved in such an effect on plant samples. However, the analysed protein preparation showed better antioxidant activity than the watermelon seed flour discussed in the work of Jaroszewska et al. [11].

### 2.4. Functional Properties

#### 2.4.1. Protein Solubility Index (PSI)

**Figure 2:**
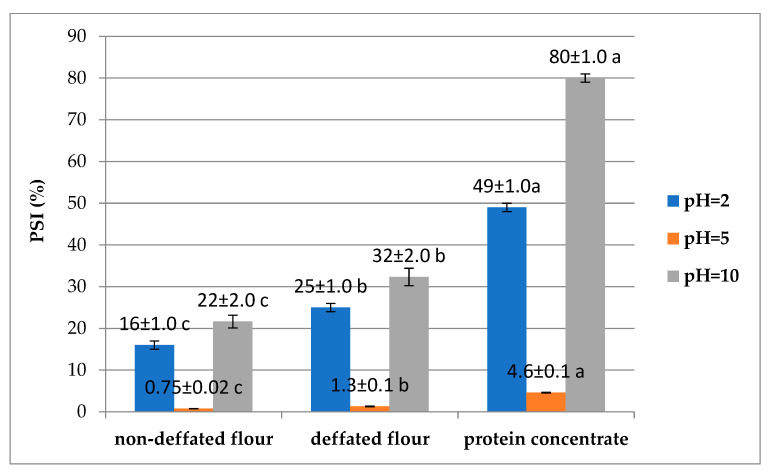
Effect of pH on protein solubility index (PSI) of watermelon seed flour and protein concentrate; the same letters within the same analysis indicate values that are not significantly different (Duncan’s test *p* ≤ 0.05 and n = 3).

Determining the functional properties of proteins is of great importance as these features reflect the interaction between components, structure, confirmation, physicochemical properties, the nature of the environment, or the food matrix [20,27]. It also helps select protein preparations with the required properties for specific food products. The functional properties of food proteins are derived from their molecular size, charge distribution and three-dimensional structure. The structure–function relationships of proteins determine how they interact with each other and other components of complex food systems [28]. Important functional properties of protein in food include hydration, water and fat binding, gelation, emulsification, foaming, and rheological properties. These properties are also influenced by environmental factors and processing conditions [29]. Figure 2 represents the solubility of nitrogen from the protein of the analysed samples at pH = 2, 5 and 10. The lowest solubility, regardless of the analysed sample, was observed at pH = 5, which is close to the isoelectric point of proteins, and the highest at an alkaline pH (pH = 10). The watermelon seed flour analysed in this study demonstrated poor solubility across different pH conditions, with protein solubility index (PSI) values below 30% (Figure 2). Notably, defatted flour showed a statistically higher PSI than non-defatted flour. In an acidic medium, the maximum solubility was lower than 40%, and in an alkaline medium, it was 80%. These results align with those reported by Lakshmi and Kaul [20], who extracted proteins from watermelon seeds using water. Compared to soy and peanut flour, watermelon seed proteins exhibit a broader solubility range. The defatting process using chemical reagents can also contribute to structural changes in proteins, thereby reducing their solubility, which is why defatted flour is characterised by the lowest protein solubility. Chemical solvents, such as n-hexane used in this study, can lead to damage to the secondary structure of proteins, changing their solubility due to the formation of protein aggregates. The solubility of protein concentrate is greatest in an alkaline environment because proteins gain a negative net charge in alkaline conditions. This negative charge causes proteins to interact more favourably with water molecules, increasing solubility. Essentially, the electrostatic repulsion between protein molecules is reduced, allowing them to dissolve more easily in water. Given the good solubility of watermelon seed protein concentrate in an alkaline environment, it can be used in bakery products as an ingredient in bread, cookies, and other baked goods to increase protein content and improve the texture, moisture retention, and overall quality or as a substitute for dairy products (in the production of plant-based alternatives to milk and yoghurt).

#### 2.4.2. Water- and Oil-Absorption Capacity

Data on selected functional properties of the tested watermelon seed flour and the obtained protein preparation are presented in Table 4. No statistical differences were found in the water (WBC) and oil (OAC) absorption of the analysed samples. The flours were characterised by higher WBC and OAC values, which may be related to their higher porosity, fibre content, starch, or, in the case of raw flour, a high lipid content [30]. In addition, the analysis of the functional properties of the obtained watermelon seed protein concentrate shows certain limitations. Low water absorption (2.46 g water/g preparation) and oil absorption (2.10 mL oil/g preparation) may limit the use of the concentrate in some types of food, especially where high values of water- and fat-binding properties are desired—in baked goods or meat-like products. This is because, in some cases, the ability of ingredients to absorb water or oil can influence the product’s structure and texture. For example, in baking, ingredients like flour or protein isolates typically absorb water, which is necessary for binding the dough and forming the desired texture. If a protein or flour has low absorption capacity, it might not bind well with water or oil, leading to a weaker structure in the final product. This could result in baked goods that are too crumbly or which do not hold together well. The observed low-water absorption of the watermelon seed protein concentrate is similar to that of pumpkin seed protein at 2.54 g/g [31] and is correlated with the high solubility of these proteins, especially in an alkaline environment (Figure 2). The oil absorption of the protein concentrate is higher than the OAC of the pumpkin seed protein isolate (0.97 mL/g), which indicates that this protein is characterised by high density and particle size, and, therefore, it absorbs less oil [31]. Due to their poor water- and oil-absorbing properties, protein preparations from watermelon seeds can be used in low-fat snacks, such as health bars, which are a substitute for sweets with a high fat and sugar content.

#### 2.4.3. Foaming Properties

Statistical differentiation was observed in the case of determining the foamability of the samples (Table 4). Non-defatted flour showed the highest foam capacity (FC) (6.83%) compared to defatted flour (0.86%). The obtained protein concentrate from watermelon seeds was characterised by FC at the level of 1.51%. The low value of foaming properties observed in this study is consistent with the results of Atuonwu and Akobundu [32], who studied proteins from Cucurbita pepo seeds. They showed that the flour from these seeds has a better foaming capacity (20%) and stability (37.5%) than the concentrate and protein isolate, resulting in higher solubility and protein elasticity. Foaming capacity increases at pH 2.0 because the protein charge increases, facilitating their spread at the phase boundary. Foam stability reaches a maximum at pH 4.0, especially in the flour and isolate, which promotes the formation of stable molecular layers. Additionally, sodium chloride concentrations in the 0.4–1.0% range affect foaming ability and stability, but CPI (*Cucurbita pepo* protein isolate) shows instability at concentrations of 0.2–1.0% [32]. However, the protein preparation obtained in the study by Lakshmi and Kaul [20] using water at pH = 11 showed better water absorption, oil absorption, and foaming properties. This may be due to several factors, such as the protein structure, solubility, and extraction method. Non-defatted flour has the highest FC values because it contains more proteins and lipids, which can support the formation of stable protein films around air bubbles. Lipids can improve the elasticity and cohesion of the film, which is crucial for good foaming ability. The extraction method also plays a role; different methods can affect protein denaturation and their functional properties. For example, proteins subjected to milder extraction processes may retain their structures and properties better than those that have been severely denatured. The lack of foam stability (FS) in samples may be due to a loss of fluids due to foam destabilisation, known as ‘leakage’. Foam stability requires a thick, flexible, and cohesive protein layer to form at the air–water interface surrounding each gas bubble. If the proteins cannot form such a structure or are not flexible enough, foam stability will be poor.

#### 2.4.4. Emulsifying Properties

**Figure 3:**
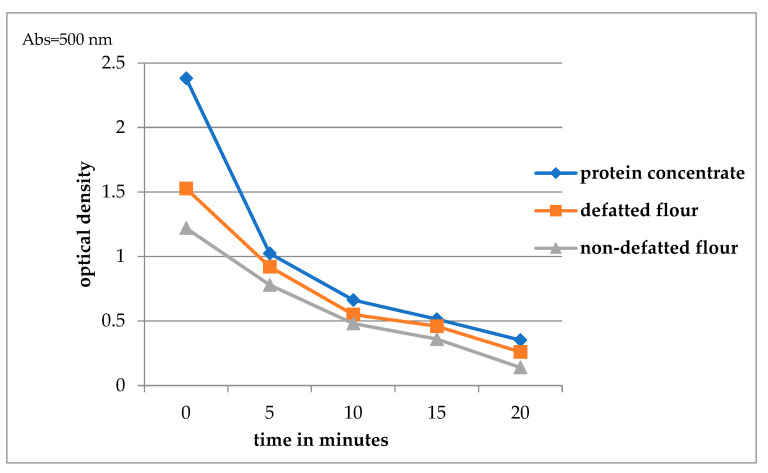
Emulsifying properties of watermelon seed flour and protein concentrate.

The ability of a protein to form an emulsion is related to its ability to absorb and stabilise the oil–water interface. The emulsion activity of the components is presented in Figure 3. The protein concentrate showed higher activity than non-defatted and defatted flour, which can be attributed to the protein concentration. The activity decreased significantly after 5 min. The emulsion stability of the protein remained the same regardless of the protein concentration and matrix (from 20 min) and should be related to the stability of the interphase area. Defatting, by removing lipid compounds, increases the contact surface of proteins with the water phase, thus increasing their emulsifying capacity. Also, greater particle size distribution in the concentrate than in flour affects the increased emulsification and stability of the emulsion due to the better distribution of proteins on the surface of the phase interface [30].

### 2.5. Colour

Of the three preparations analysed, defatted flour was the lightest colour (L = 63.50), non-defatted flour was characterised by a darker colour at L = 55.69, and the protein concentrate was characterised by a lightness level similar to defatted flour and was L = 61.28 (Table 5). Additionally, differences in a* and b* values can be seen when analysing other colour parameters. Non-defatted flour had a* = 4.62, which suggests that it is redder than the other preparations. Defatted flour with a* = 4.08 and protein concentrate with an a* value of 4.21 were darker in this range. The b* value for the non-defatted flour was 12.45, which meant that it had a more pronounced yellow hue than the defatted flour (b* = 13.82) and the protein concentrate (b* = 14.87), suggesting that both of the latter preparations have a more intense yellow colour. It is also worth noting the chromatic value (C*), which for the non-defatted flour was 13.28, making it the darkest preparation in this analysis. The defatted flour with a C* value of 14.41 and the protein concentrate with a C* value of 15.45 indicate their more intense colour, which may be important for their visual context and potential use in food products. Finally, in the context of colour, it can be seen that all preparations differ from each other, which may affect their usefulness both in the food industry and in dietary supplementation. The analysed results suggest that defatted flour and protein concentrate may be more visually attractive than non-defatted flour, which may influence consumer choice.

## 3. Materials and Methods

### 3.1. Materials and Chemicals

Commercial watermelon seed flour (*Citrullus lanatus* L.) was purchased from the Ol’Vita company (Wrocław, Poland). The raw material was sieved in order to standardise its granulation. All chemicals used in the experiment were of an analytical grade.

### 3.2. Preparation of Defatted Flour

To obtain the defatted flour from watermelon seeds, the flour was mixed with n-hexane in a 1:10 ratio, at room temperature, for 90 min, by continuous stirring (with the use of a magnetic stirrer: IKA, Staufen im Breisgau, Germany). After the defatting process, the solvent was separated from the flour and left under a fume cupboard for about 12 h to evaporate the remaining solvent.

### 3.3. Preparation of Protein Concentrate

Protein preparation was prepared according to the method of [21] with the following modifications. Defatted flour was mixed with a 1.2% NaOH solution at a ratio of 1:50. The slurry was stirred for 30 min at 40 °C and then centrifuged at 4000× *g* for 15 min (MPW-380, Heraeus Sepatech, Osteorode, Germany). The supernatant was collected, and the coagulation of dissolved proteins was performed at the isoelectric point (pH = 4.5) using 2 M HCl. To separate the coagulated proteins, the mixture was centrifuged at 4000× *g* for 15 min (MPW-380, Heraeus Sepatech, Osteorode, Germany), the obtained supernatant was decanted, and the collected precipitate was neutralised with the use of 0.1 M NaOH before being washed and freeze-dried using the Christ Alpha 1–4 LSCplus lyophiliser (Osterode am Hatz, Germany), and then the preparation was sieved and stored at −20 °C until further analysis.

### 3.4. Chemical Composition

The dry matter (DM) and ash content were measured by the constant mass method [33]. The moisture content of the analysed samples was determined on the basis of weight loss during thermal drying at 105 °C until a constant weight was achieved. The total ash content was determined by adding 1 g of the protein preparation to a crucible, incinerating it in a muffle furnace at 550 °C, and determining the weight of the residue. The total protein content was calculated from the amount of nitrogen by a 6.25 factor as a standard and evaluated according to the Kjeldahl method using a Büchi Distillation Unit K-355 (Athens, Greece). Fat content was determined according to the standard method [33] by means of the Soxhlet method in a Büchi B-811 apparatus (Flawil, Switzerland) with the use of diethyl ether after hydrolysis of the sample with 4 N HCl. Total carbohydrates were calculated using a difference calculation (100—the sum of protein, fat, ash and moisture). Data are reported as mean values ± standard deviation (SD) for three measurements.

### 3.5. Amino Acid Composition

The analysed samples were acid-hydrolysed [34] and placed in an AAA400 automatic amino acid analyser (INGOS s.r.o., Prague, Czech Republic). A two-wavelength photometer (440 and 570 nm) was employed as a detector. The length of the column packed with ion exchanger Ostion LG ANB (INGOS s.r.o., Prague, Czech Republic) was 350 × 3.7 mm, and the column temperature was maintained at 40–70 °C, and the detector temperature was kept at 121 °C. The amino acids were quantified using the ninhydrin method. Glutamine and asparagine were expressed as glutamic acid and aspartic acid, respectively. No analysis was carried out for tryptophan. Calculations were performed with the computer program Chromulan (Pikron s.r.o., Prague, Czech Republic). All amino acid profiles were analysed in duplicate.

### 3.6. Computation of Nutritional Indices

The analysed data were used for computing the nutritional indices as the chemical score (CS) and essential amino acid index (EAAI) by comparing the amino acid composition of the test samples with that suggested by FAO/WHO/UNU [35] for growing children and adults.

### 3.7. Total Polyphenolic Compounds and Antioxidant Activity

Extraction procedure: The material (~1 g) was mixed with 10 mL of 80% MeOH in deionised water with 1% HCl. Then, samples were sonicated twice (800 W, 40 Hz, Sonic 6D, Polsonic, Warsaw, Poland) for 20 min at 20 °C and left for 24 h at 4 °C. After this procedure, the extract was centrifuged for 10 min at 19,000× *g*, and the supernatant was filtered through a hydrophilic PTFE with a 0.20 µm membrane (Millex Samplicity Filter, Merck, Darmstadt, Germany) and used for tests.

Total polyphenolic compounds: Total polyphenols were measured using the Folin–Ciocalteu method [36]. Briefly, the extract (100 µL) was mixed with distilled water (2000 µL), the Folin–Ciocalteu phenol reagent (200 µL) and 20% sodium carbonate solution in water (1000 µL). The sample was incubated for 1 h in the dark at 20 °C. The absorbance was measured at 765 nm (UV-2401 PC, Shimadzu Corp., Kyoto, Japan). Total polyphenols were calculated based on the curve equation y = 23.35 × Abs − 1.675 and presented in mg of gallic acid equivalents (GAE)/g DM).

Antioxidant activity: The procedure for the determination of antiradical activity, using 2,2′-azino-bis(3-ethylbenzothiazoline-6-sulfonic acid) diammonium salt (ABTS) is described in Ref [37]. Briefly, 30 µL of the sample was mixed with 3 mL of the ABTS reagent. After 6 min of the reaction, the absorbance was measured at 734 nm (UV-2401 PC spectrophotometer; Shimadzu, Kyoto, Japan). The antioxidant activity was presented in µmol of Trolox (TE)/g DM.

### 3.8. Functional Properties

#### 3.8.1. Protein Solubility Index

The pH–solubility profile index (PSI) at different levels of pH (2, 5 and 10) for the watermelon flour and protein preparation was determined according to the method of [38], with slight modifications. Briefly, 200 mg of the sample was weighed in the tube, 15 mL of distilled water was added, and then the pH was adjusted (2, 5 or 10) using either 0.5 M NaOH or 0.5 M HCl. The protein solutions were shaken at room temperature for 30 min and successively centrifuged at 4000× *g* for 15 min (Rotofix 32A, Hettich, Tuttlingen, Germany). The protein content of the supernatants was determined by the Lowry method. Protein solubility was calculated as follows:

PSI = (PCS/TPC) × 100 (%)(1)

where PCS is the protein content in the supernatant after centrifugation, and TPC is the total protein content present in the protein sample. PSI at different levels of pH were determined in three analytical repetitions.

#### 3.8.2. Water- and Oil-Absorption Capacity

The water-binding capacity (WBC) of the watermelon flour and protein preparation was determined according to the method described by [38] with slight modifications. To the previously weighed falcon, 1 g of the powdered sample was weighed in a test tube containing 20 mL of distilled water. The mixture was shaken for 15 min and then again for one minute. This slurry was centrifuged at 4000× *g* for 15 min (Rotofix 32A, Hettich, Tuttlingen, Germany). The separated solid was oven-dried at 50 °C for 30 min. WBC was expressed as the amount of water (g) absorbed by 1 g of the preparation and by 1 g of the protein. WBC was determined in three analytical repetitions.

The oil-absorption capacity (OAC) of the watermelon flour and protein preparation was determined using the method described in Ref [39], with slight modifications. Briefly, 1 g of the sample was weighed in the test tube and mixed with 25 mL of rapeseed oil using a multi-station shaker for 30 min. The resulting protein–oil mixture was separated using a centrifuge (4000× *g*; Rotofix 32A, Hettich, Tuttlingen, Germany) for 10 min. Immediately after centrifugation, the supernatant was carefully poured into a 30 mL graduated cylinder, and the volumes were recorded. OAC was expressed as the amount of oil (mL) absorbed by 1 g of the preparation and by 1 g of protein. OAC was determined in three analytical repetitions.

#### 3.8.3. Foaming Properties

The foam capacity (FC) and stability (FS) of the watermelon flour and protein preparation were measured according to the method of [40], with slight modifications. Briefly, 0.25 g of the sample was weighed into the tube, and 50 mL of distilled water was added to it. The sample was then homogenised for 2 min at 16,000 rpm (T25 basic ULTRA-TURRAX^®^; IKA Werke, Staufen im Breisgau, Germany). The beaten sample was immediately transferred to a measuring cylinder where the total foam volume was determined after 0, 5, 10, 30, and 60 min. FC and FS were calculated according to the following equations:

FC = (VA/VB) × 100 (%)(2)

where VA denotes the volume after whipping (mL) and VB is the volume before whipping (mL).

FS = (VC/VT) × 100 (%)(3)

where VC denotes the volume before whipping (cm^3^), and VT is the volume after a certain time (mL). Foaming properties were determined in three analytical repetitions. No foam stability was observed in any of the tested samples.

#### 3.8.4. Emulsifying Properties

The emulsification activity index (EAI) and stability index (ESI) were estimated using the method described by [41] with slight modifications. Briefly, 0.1 g of the watermelon flour and protein preparation was homogenised with 2 mL of oil for 1 min using a T25 basic ULTRA-TURRAX^®^ homogeniser (IKA-Werke GmbH & Co. KG, Staufen im Breisgau, Germany). The pH was maintained at 7 using NaOH i HCl. Samples of 50 µL of the prepared emulsion were pipetted from the bottom of the container at 0, 5, 15, and 20 min after homogenisation and added to 5 mL of the 0.1% sodium dodecyl sulphate (SDS) solution and mixed by gentle shaking. The absorbance of the diluted solution was measured at 500 nm using a spectrophotometer (UV-2401 PC, Shimadzu Corp., Kyoto, Japan). EAI can be calculated as follows:

(4) $$EAI(\frac{m2}{g})=\frac{2\times 2303\times A0\times D}{\emptyset \times c\times 10,000}$$

where A0 is the absorbance at 500 nm immediately following homogenisation, D is the dilution factor, Ø is the oil volume fraction, and c is the protein concentration (g/mL).

ESI can be calculated as follows:

ESI (min) = A0 × ΔT/ΔA(5)

where ΔT is equal to 5, 10, 15, and 20 min and ΔA is the difference in absorbance at 500 nm after 0, 5, 10, 15 and 20 min. Measurements were performed in triplicates.

### 3.9. Colour Determination

Colour measurements of the flour and protein concentrate were determined using a Konica Minolta CM-5 spectrophotometer (Tokyo, Japan), illuminated under standardised conditions (standard light D65). Colour data are provided as the CIE L* (lightness) coordinate, which varies in the range 0–100, a* (redness–greenness) where negative values are represented by a larger share of redness in the colour, and positive values are presented by a larger share of greenness in the colour, b* (yellowness–blueness) where negative values are represented by a larger share of yellowness in the colour, and positive values mean the share of blueness in the colour values. The instrument was calibrated against a white calibration plate. Each sample was determined in five analytical repetitions, and the read values of L*, a*, and b* were averaged. Hue angle (h*) and chroma (C*) colour space parameters were calculated from the a* and b* values [42]:

(6) $$Hue\;angle=Arctan\frac{b*}{a*}$$

(7) $$Chroma=\sqrt{{(a*}^{2})+{(b*}^{2})}$$

Chroma describes the intensity or purity of the colour and answers whether the colour is vivid or grey (*C* = 0 colour is neutral, *C* > O colour is more intense, saturated). Hue determines what type of colour it is, based on two colour coordinates, a* and b*, and allows for a comparison of the shades of different samples.

### 3.10. Statistical Analysis

Statistical analysis of all data was performed using one-way analysis of variance (ANOVA). The analysis of the chemical composition and functional properties was performed in triplicate. Duncan’s range test was used to determine the differences among the samples with a probability level of 0.05. Statistical analysis and standard deviations were determined using Statistica v. 13.3 software (Dell Software Inc., Round Rock, TX, USA).

## 4. Conclusions

The novelty of this study lies in its exploration of watermelon seed protein concentrate as a sustainable plant-based protein source, particularly from a food industry byproduct. In the present study, a watermelon seed protein concentrate was produced using the alkaline extraction method and isoelectric point coagulation. The study provides valuable insights into the protein content, functional properties, and antioxidant activity of the concentrate. The resulting protein preparation displayed good nutritional and functional properties, making it a highly valuable plant-based ingredient. The amino acid profile of watermelon seed protein was rich in essential amino acids, particularly arginine, glutamic acid, and aspartic acid, which can offer health benefits, including therapeutic effects. The protein concentrate demonstrated improved nutritional quality, as reflected by its higher essential amino acid index compared to non-defatted and defatted flours. In terms of antioxidant potential, despite there being no significant differences in total phenolic content, the protein concentrate exhibited the highest antioxidant activity, making it a valuable functional ingredient with health-promoting properties. Its high protein composition, coupled with antioxidant activity, positions it as a versatile addition to the growing market for plant-based foods. Despite having low water and oil absorption capacity, low emulsification and foaming properties, its good protein solubility generally in an alkaline environment makes it suitable for applications in baked goods and plant-based dairy alternatives. Overall, watermelon seed protein concentrate and defatted flour offer promising alternatives for enriching the protein content of food products while providing additional functional benefits. Their unique amino acid composition, antioxidant activity, and favourable colour properties make them suitable for use in a wide range of food formulations, particularly in health-conscious, plant-based diets. Further exploration of their applications, especially in the food industry, could provide new opportunities for utilising watermelon seeds as a sustainable and nutritious ingredient.

## Figures and Tables

**Table 1:** Chemical compositions (g/100 g) of watermelon seed flour and protein concentrate.

Analysed Material	Dry Matter	Total Protein	Ash	Fat	Carbohydrates
Non-defatted flour	95.75 ± 0.02 ^a^	32.08 ± 0.01 ^c^	3.53 ± 0.04 ^a^	6.83 ± 0.07 ^a^	53.31 ± 0.06 ^b^
Defatted flour	95.26 ± 0.05 ^a^	34.71 ± 0.02 ^b^	3.67 ± 0.05 ^a^	0.86 ± 0.08 ^c^	56.02 ± 0.10 ^a^
Protein concentrate	95.16 ± 0.05 ^a^	82.52 ± 0.29 ^a^	2.50 ±0.2 ^b^	1.51 ± 0.03 ^b^	8.63 ± 0.41 ^c^

Values are means ± standard deviation. n = 3; ^a–c^ the same letters in the columns mean homogeneous groups (Duncan’s test *p* ≤ 0.05).

**Table 2:** Amino acid profile (mg/g) of watermelon seed flour and protein concentrate.

Amino Acid	Non-Defatted Flour	Defatted Flour	Protein Concentrate
IAA *			
Leucine	13.89 ± 0.07 ^b^	13.94 ± 0.29 ^b^	50.52 ± 0.61 ^a^
Isoleucine	7.65 ± 0.07 ^b^	7.61 ± 0.23 ^b^	29.00 ± 0.56 ^a^
Methionine	0.87 ± 0.02 ^b^	0.62 ± 0.01 ^b^	19.64 ± 0.30 ^a^
Cysteine	1.18 ± 0.03 ^b^	1.19 ± 0.08 ^b^	1.65 ± 0.21 ^a^
Phenylalanine	10.47 ± 0.16 ^b^	10.41 ± 0.27 ^b^	43.01 ± 0.54 ^a^
Threonine	6.94 ± 0.15 ^b^	7.11 ± 0.27 ^b^	24.39 ± 0.47 ^a^
Lysine	6.31 ± 0.08 ^b^	6.28 ± 0.21 ^b^	18.00 ± 0.30 ^a^
Tyrosine	3.38 ± 0.06 ^b^	3.00 ± 0.07 ^b^	22.17 ± 0.42 ^a^
Valine	9.04 ± 0.04 ^b^	9.19 ± 0.23 ^b^	33.42 ± 0.52 ^a^
DAA **			
Aspartic acid	18.57 ± 0.11 ^b^	19.14 ± 0.15 ^b^	71.71 g ± 1.22 ^a^
Glutamic acid	54.76 ± 1.21 ^b^	54.98 ± 1.66 ^b^	184.83 ± 4.75 ^a^
Serine	9.51 ± 0.01 ^b^	9.56 ± 0.41 ^b^	33.20 ± 0.90 ^a^
Glycine	12.38 ± 0.08 ^b^	12.27 ± 0.27 ^b^	33.99 ± 0.44 ^a^
Alanine	9.69 ± 0.04 ^b^	9.73 ± 0.35 ^b^	32.42 ± 0.45 ^a^
Histidine	4.79 ± 0.28 ^b^	4.65 ± 0.42 ^b^	16.17 ± 0.37 ^a^
Arginine	26.04 ± 0.17 ^b^	25.01 ± 0.35 ^b^	99.73 ± 1.63 ^a^
Proline	6.86 ±0.30 ^a^	7.65 ± 0.69 ^a^	16.89 ± 7.65 ^a^
Total amino acids	202.29 ± 1.82 ^b^	202.06 ± 5.94 ^b^	730.69 ± 21.34 ^a^

Values are means ± standard deviation. n = 2; ^a–b^ the same letters in verse mean homogenous groups (Duncan’s test *p* ≤ 0.05); IAA *— indispensable amino acids; DAA **—dispensable amino acids.

**Table 3:** Mean values of CS and EAAI of watermelon seed flour and protein concentrate.

Coefficient	Amino Acid	Non-Defatted Flour	Defatted Flour	Protein Concentrate
			%	
CS	Leu	73.39	68.14	103.73
	Ile	79.33	73.00	117.00
	Met + Cys	29.09 *	23.64 *	117.27
	Phe + Tyr	142.63	132.89	215.00
	Thr	45.65	37.39 **	116.96
	Lys	43.78 **	40.22	48.44 *
	Val	72.31	67.95	103.85
EAAI		63.18	56.69	109.46

* First limiting amino acid; ** second limiting amino acid. CS—chemical score, EAAI—essential amino acid index.

**Table 4:** Functional properties of watermelon seed flour and protein concentrate.

Functional Property	Non-Defatted Flour	Defatted Flour	Protein Concentrate
WBC (g water/g preparation)	2.51 ± 0.03 ^a^	2.50 ± 0.16 ^a^	2.46 ± 0.08 ^a^
WBC (g water/g protein)	0.81 ± 0.03 ^a^	0.87 ± 0.16 ^a^	2.03 ± 0.08 ^a^
OAC (ml oil/g preparation)	2.27 ± 0.54 ^a^	1.29 ± 0.61 ^a^	2.10 ± 0.27 ^a^
OAC (ml oil/g protein)	0.73 ± 0.54 ^a^	0.45 ± 0.61 ^a^	1.73 ± 0.27 ^a^
FC (%)	6.83 ± 0.07 ^a^	0.86 ± 0.08 ^c^	1.51 ± 0.03 ^b^

Values are means ± standard deviation. n = 3; ^a–c^ the same letters in the columns mean homogeneous groups (Duncan’s test *p* ≤ 0.05). WBC—water-binding capacity; OAC—oil-absorption capacity; FC—foam capacity.

**Table 5:** Colour of watermelon seed flours and protein concentrate.

Analysed Material	L*	a*	b*	C*	h
Non-defatted flour	55.69 ± 0.45 ^c^	4.62 ± 0.10 ^a^	12.45 ± 0.26 ^c^	13.28 ± 0.27 ^c^	69.64 ± 0.32 ^c^
Defatted flour	63.50 ± 0.33 ^a^	4.08 ± 0.08 ^c^	13.82 ± 0.15 ^b^	14.41 ± 0.16 ^b^	73.56 ± 0.21 ^b^
Protein concentrate	61.28 ± 0.38 ^b^	4.21 ± 0.06 ^b^	14.87 ± 0.18 ^a^	15.45 ± 0.18 ^a^	74.19 ± 0.23 ^a^

Values are means ± standard deviation. n = 5; ^a–c^ the same letters in columns mean homogeneous groups (Duncan’s test *p* ≤ 0.05). L*—lightness; a*—redness to greenness; b*—yellowness to blueness; C*—chroma colour; h—hue angel.

## Data Availability

The data are contained within the article.
